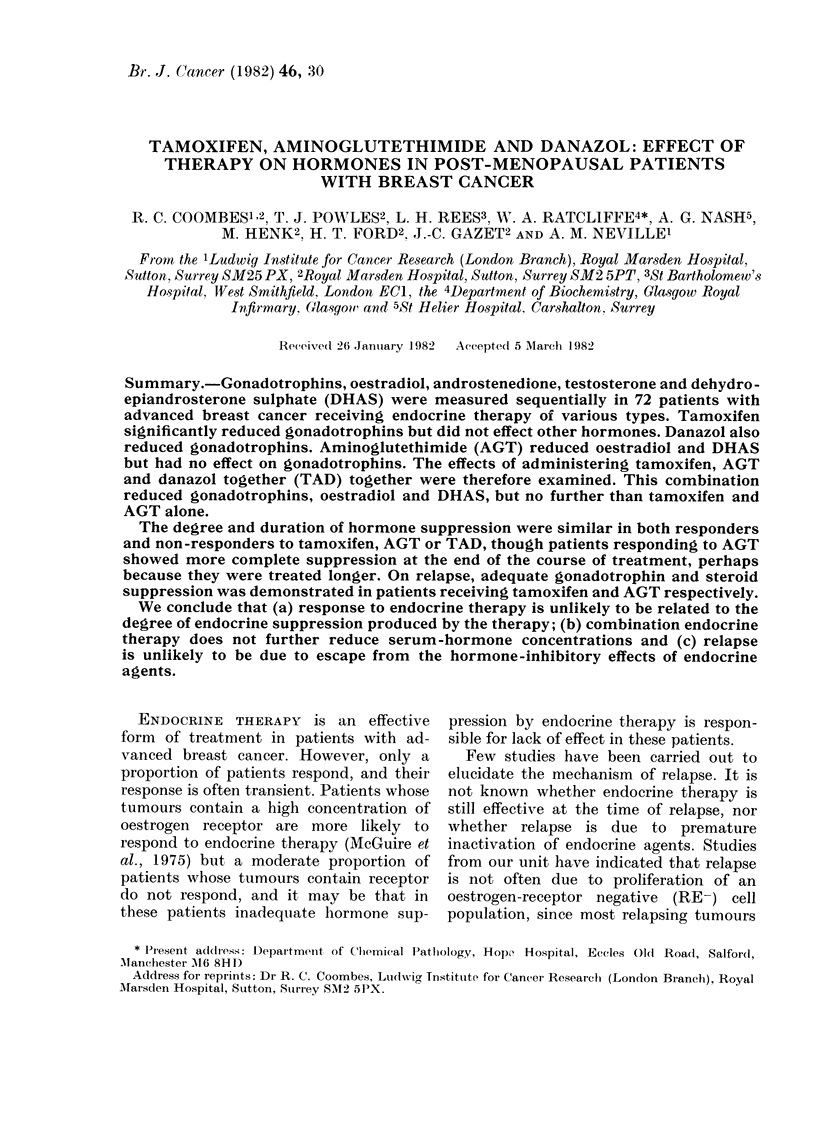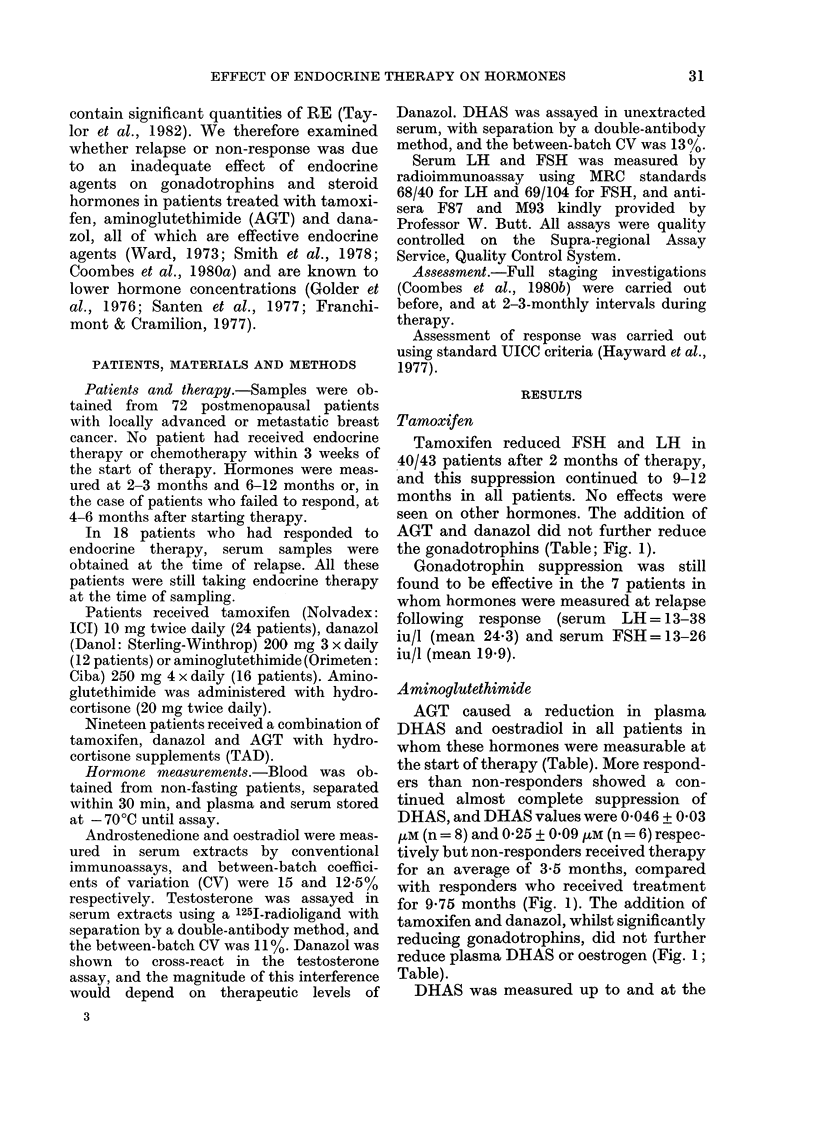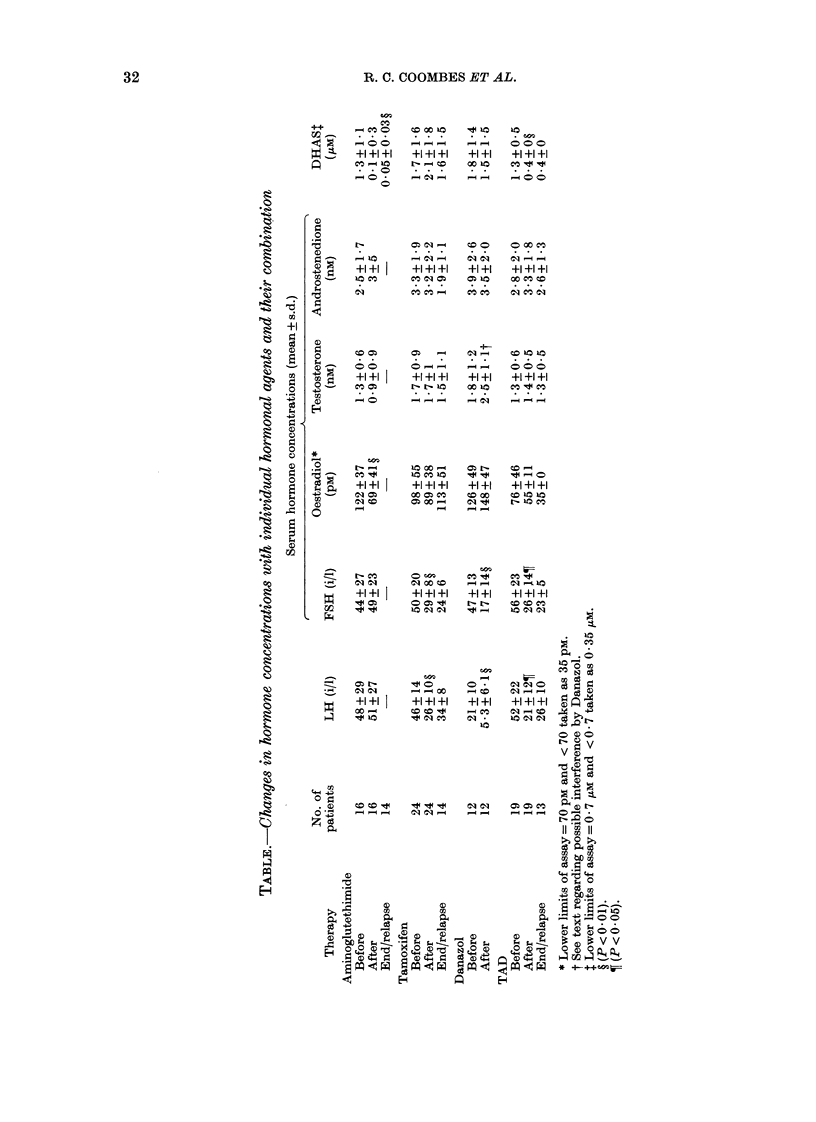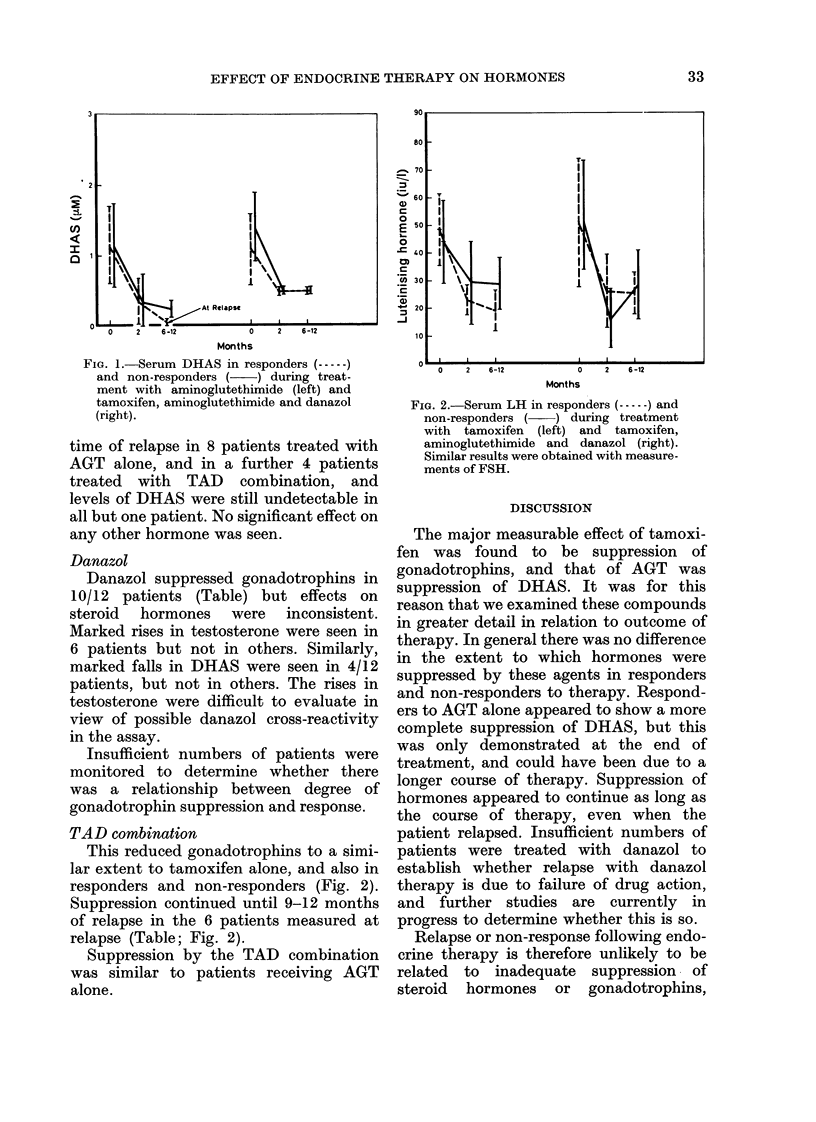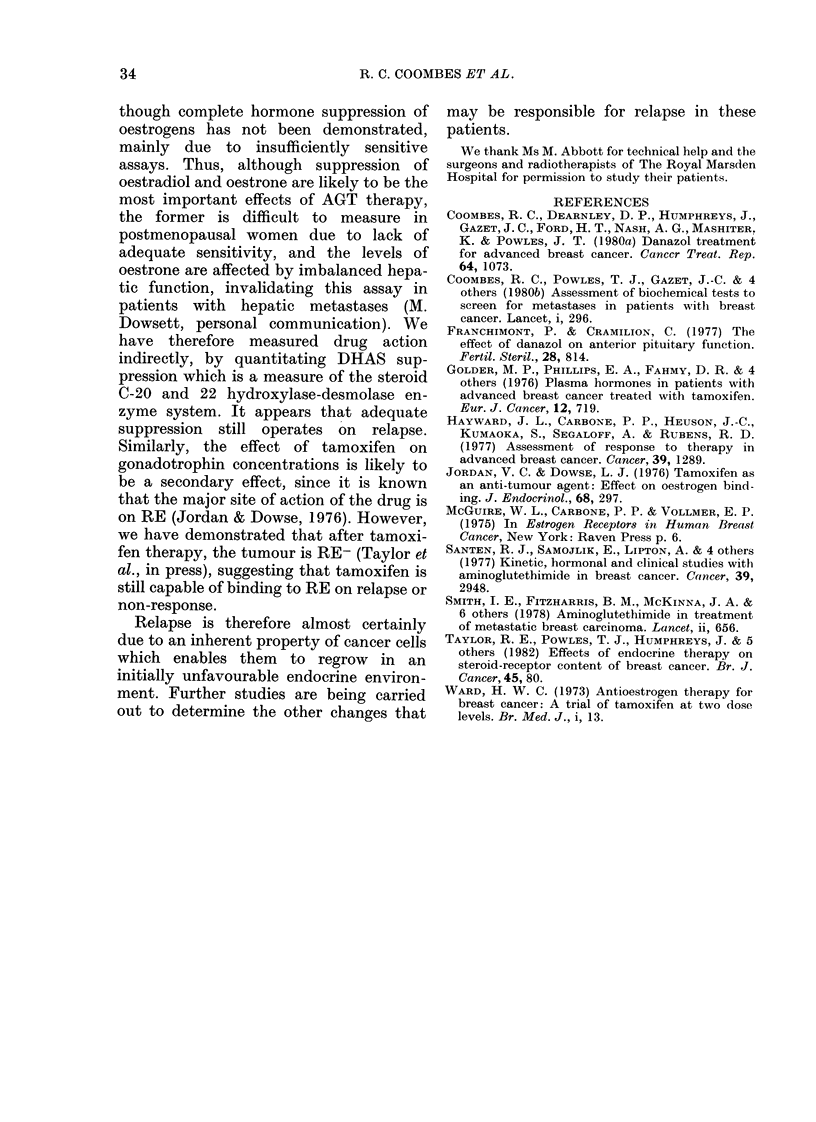# Tamoxifen, aminoglutethimide and danazol: effect of therapy on hormones in post-menopausal patients with breast cancer.

**DOI:** 10.1038/bjc.1982.161

**Published:** 1982-07

**Authors:** R. C. Coombes, T. J. Powles, L. H. Rees, W. A. Ratcliffe, A. G. Nash, M. Henk, H. T. Ford, J. C. Gazet, A. M. Neville

## Abstract

Gonadotrophins, oestradiol, androstenedione, testosterone and dehydroepiandrosterone sulphate (DHAS) were measured sequentially in 72 patients with advanced breast cancer receiving endocrine therapy of various types. Tamoxifen significantly reduced gonadotrophins but did not effect other hormones. Danazol also reduced gonadotrophins. Aminoglutethimide (AGT) reduced oestradiol and DHAS but had not effect on gonadotrophins. The effects of administering tamoxifen, AGT and danazol together (TAD) together were therefore examined. This combination reduced gonadotrophins, oestradiol and DHAS, but no further than tamoxifen and AGT alone. The degree and duration of hormone suppression were similar in both responders and non-responders to tamoxifen, AGT or TAD, though patients responding to AGT showed more complete suppression at the end of the course of treatment, perhaps because they were treated longer. On relapse, adequate gonadotrophin and steroid suppression was demonstrated in patients receiving tamoxifen and AGT respectively. We conclude that (a) response to endocrine therapy is unlikely to be related to the degree of endocrine suppression produced by the therapy; (b) combination endocrine therapy does not further reduce serum-hormone concentrations and (c) relapse is unlikely to be due to escape from the hormone-inhibitory effects of endocrine agents.


*Br. J. Cancer* (1982) **46**, 30

### TAMOXIFEN, AMINOGLUTETHIMIDE AND DANAZOL: EFFECT OF THERAPY ON HORMONES IN POST-MENOPAUSAL PATIENTS WITH BREAST CANCER

R. C. COOMBES[1,2], T. J. POWLES[2], L. H. REES[3], W. A. RATCLIFFE[4]*, A. G. NASH[5], M. HENK[2], H. T. FORD[2], J.-C. GAZET[2] AND A. M. NEVILLE[1]

*From the [1]Ludwig Institute for Cancer Research (London Branch), Royal Marsden Hospital, Sutton, Surrey SM25PX, [2]Royal Marsden Hospital, Sutton, Surrey SM2 5PT, [3]St Bartholomew's Hospital, West Smithfield, London EC1, the [4]Department of Biochemistry, Glasgow Royal Infirmary, Glasgow and [5]St Helier Hospital, Carshalton, Surrey*

Received 26 January 1982 Accepted 5 March 1982

**Summary.—Gonadotrophins, oestradiol, androstenedione, testosterone and dehydroepiandrosterone sulphate (DHAS) were measured sequentially in 72 patients with advanced breast cancer receiving endocrine therapy of various types. Tamoxifen significantly reduced gonadotrophins but did not effect other hormones. Danazol also reduced gonadotrophins. Aminoglutethimide (AGT) reduced oestradiol and DHAS but had no effect on gonadotrophins. The effects of administering tamoxifen, AGT and danazol together (TAD) together were therefore examined. This combination reduced gonadotrophins, oestradiol and DHAS, but no further than tamoxifen and AGT alone.**

**The degree and duration of hormone suppression were similar in both responders and non-responders to tamoxifen, AGT or TAD, though patients responding to AGT showed more complete suppression at the end of the course of treatment, perhaps because they were treated longer. On relapse, adequate gonadotrophin and steroid suppression was demonstrated in patients receiving tamoxifen and AGT respectively.**

**We conclude that (a) response to endocrine therapy is unlikely to be related to the degree of endocrine suppression produced by the therapy; (b) combination endocrine therapy does not further reduce serum-hormone concentrations and (c) relapse is unlikely to be due to escape from the hormone-inhibitory effects of endocrine agents.**

ENDOCRINE THERAPY is an effective form of treatment in patients with advanced breast cancer. However, only a proportion of patients respond, and their response is often transient. Patients whose tumours contain a high concentration of oestrogen receptor are more likely to respond to endocrine therapy (McGuire *et al.*, 1975) but a moderate proportion of patients whose tumours contain receptor do not respond, and it may be that in these patients inadequate hormone suppression by endocrine therapy is responsible for lack of effect in these patients.

Few studies have been carried out to elucidate the mechanism of relapse. It is not known whether endocrine therapy is still effective at the time of relapse, nor whether relapse is due to premature inactivation of endocrine agents. Studies from our unit have indicated that relapse is not often due to proliferation of an oestrogen-receptor negative ($RE^-$) cell population, since most relapsing tumours

* Present address: Department of Chemical Pathology, Hope Hospital, Eccles Old Road, Salford, Manchester M6 8HD

Address for reprints: Dr R. C. Coombes, Ludwig Institute for Cancer Research (London Branch), Royal Marsden Hospital, Sutton, Surrey SM2 5PX.

contain significant quantities of RE (Taylor *et al.*, 1982). We therefore examined whether relapse or non-response was due to an inadequate effect of endocrine agents on gonadotrophins and steroid hormones in patients treated with tamoxifen, aminoglutethimide (AGT) and danazol, all of which are effective endocrine agents (Ward, 1973; Smith *et al.*, 1978; Coombes *et al.*, 1980*a*) and are known to lower hormone concentrations (Golder *et al.*, 1976; Santen *et al.*, 1977; Franchimont & Cramilion, 1977).

### PATIENTS, MATERIALS AND METHODS

*Patients and therapy.*—Samples were obtained from 72 postmenopausal patients with locally advanced or metastatic breast cancer. No patient had received endocrine therapy or chemotherapy within 3 weeks of the start of therapy. Hormones were measured at 2–3 months and 6–12 months or, in the case of patients who failed to respond, at 4–6 months after starting therapy.

In 18 patients who had responded to endocrine therapy, serum samples were obtained at the time of relapse. All these patients were still taking endocrine therapy at the time of sampling.

Patients received tamoxifen (Nolvadex: ICI) 10 mg twice daily (24 patients), danazol (Danol: Sterling-Winthrop) 200 mg 3 × daily (12 patients) or aminoglutethimide (Orimeten: Ciba) 250 mg 4 × daily (16 patients). Aminoglutethimide was administered with hydrocortisone (20 mg twice daily).

Nineteen patients received a combination of tamoxifen, danazol and AGT with hydrocortisone supplements (TAD).

*Hormone measurements.*—Blood was obtained from non-fasting patients, separated within 30 min, and plasma and serum stored at −70°C until assay.

Androstenedione and oestradiol were measured in serum extracts by conventional immunoassays, and between-batch coefficients of variation (CV) were 15 and 12·5% respectively. Testosterone was assayed in serum extracts using a $^{125}$I-radioligand with separation by a double-antibody method, and the between-batch CV was 11%. Danazol was shown to cross-react in the testosterone assay, and the magnitude of this interference would depend on therapeutic levels of Danazol. DHAS was assayed in unextracted serum, with separation by a double-antibody method, and the between-batch CV was 13%.

Serum LH and FSH was measured by radioimmunoassay using MRC standards 68/40 for LH and 69/104 for FSH, and antisera F87 and M93 kindly provided by Professor W. Butt. All assays were quality controlled on the Supra-regional Assay Service, Quality Control System.

*Assessment.*—Full staging investigations (Coombes *et al.*, 1980*b*) were carried out before, and at 2–3-monthly intervals during therapy.

Assessment of response was carried out using standard UICC criteria (Hayward *et al.*, 1977).

### RESULTS

### *Tamoxifen*

Tamoxifen reduced FSH and LH in 40/43 patients after 2 months of therapy, and this suppression continued to 9–12 months in all patients. No effects were seen on other hormones. The addition of AGT and danazol did not further reduce the gonadotrophins (Table; Fig. 1).

Gonadotrophin suppression was still found to be effective in the 7 patients in whom hormones were measured at relapse following response (serum LH = 13–38 iu/l (mean 24·3) and serum FSH = 13–26 iu/l (mean 19·9).

### *Aminoglutethimide*

AGT caused a reduction in plasma DHAS and oestradiol in all patients in whom these hormones were measurable at the start of therapy (Table). More responders than non-responders showed a continued almost complete suppression of DHAS, and DHAS values were 0·046 ± 0·03 $\mu$M (n = 8) and 0·25 ± 0·09 $\mu$M (n = 6) respectively but non-responders received therapy for an average of 3·5 months, compared with responders who received treatment for 9·75 months (Fig. 1). The addition of tamoxifen and danazol, whilst significantly reducing gonadotrophins, did not further reduce plasma DHAS or oestrogen (Fig. 1; Table).

DHAS was measured up to and at the

TABLE.—*Changes in hormone concentrations with individual hormonal agents and their combination*

| Therapy | No. of patients | Serum hormone concentrations (mean ± s.d.): LH (i/l) | FSH (i/l) | Oestradiol* (pM) | Testosterone (nM) | Androstenedione (nM) | DHAS‡ (μM) |
|---|---|---|---|---|---|---|---|
| Aminoglutethimide | | | | | | | |
| Before | 16 | 48 ± 29 | 44 ± 27 | 122 ± 37 | 1·3 ± 0·6 | 2·5 ± 1·7 | 1·3 ± 1·1 |
| After | 16 | 51 ± 27 | 49 ± 23 | 69 ± 41§ | 0·9 ± 0·9 | 3 ± 5 | 0·1 ± 0·3 |
| End/relapse | 14 | — | — | — | — | — | 0·05 ± 0·03§ |
| Tamoxifen | | | | | | | |
| Before | 24 | 46 ± 14 | 50 ± 20 | 98 ± 55 | 1·7 ± 0·9 | 3·3 ± 1·9 | 1·7 ± 1·6 |
| After | 24 | 26 ± 10§ | 29 ± 8§ | 89 ± 38 | 1·7 ± 1 | 3·2 ± 2·2 | 2·1 ± 1·8 |
| End/relapse | 14 | 34 ± 8 | 24 ± 6 | 113 ± 51 | 1·5 ± 1·1 | 1·9 ± 1·1 | 1·6 ± 1·5 |
| Danazol | | | | | | | |
| Before | 12 | 21 ± 10 | 47 ± 13 | 126 ± 49 | 1·8 ± 1·2 | 3·9 ± 2·6 | 1·8 ± 1·4 |
| After | 12 | 5·3 ± 6·1§ | 17 ± 14§ | 148 ± 47 | 2·5 ± 1·1† | 3·5 ± 2·0 | 1·5 ± 1·5 |
| TAD | | | | | | | |
| Before | 19 | 52 ± 22 | 56 ± 23 | 76 ± 46 | 1·3 ± 0·6 | 2·8 ± 2·0 | 1·3 ± 0·5 |
| After | 19 | 21 ± 12¶ | 26 ± 14¶ | 55 ± 11 | 1·4 ± 0·5 | 3·3 ± 1·8 | 0·4 ± 0§ |
| End/relapse | 13 | 26 ± 10 | 23 ± 5 | 35 ± 0 | 1·3 ± 0·5 | 2·6 ± 1·3 | 0·4 ± 0 |

* Lower limits of assay = 70 pM and < 70 taken as 35 pM.
† See text regarding possible interference by Danazol.
‡ Lower limits of assay = 0·7 μM and < 0·7 taken as 0·35 μM.
§ ($P < 0·01$).
¶ ($P < 0·05$).

FIG. 1.—Serum DHAS in responders (-----) and non-responders (——) during treatment with aminoglutethimide (left) and tamoxifen, aminoglutethimide and danazol (right).

time of relapse in 8 patients treated with AGT alone, and in a further 4 patients treated with TAD combination, and levels of DHAS were still undetectable in all but one patient. No significant effect on any other hormone was seen.

*Danazol*

Danazol suppressed gonadotrophins in 10/12 patients (Table) but effects on steroid hormones were inconsistent. Marked rises in testosterone were seen in 6 patients but not in others. Similarly, marked falls in DHAS were seen in 4/12 patients, but not in others. The rises in testosterone were difficult to evaluate in view of possible danazol cross-reactivity in the assay.

Insufficient numbers of patients were monitored to determine whether there was a relationship between degree of gonadotrophin suppression and response.

*TAD combination*

This reduced gonadotrophins to a similar extent to tamoxifen alone, and also in responders and non-responders (Fig. 2). Suppression continued until 9–12 months of relapse in the 6 patients measured at relapse (Table; Fig. 2).

Suppression by the TAD combination was similar to patients receiving AGT alone.

FIG. 2.—Serum LH in responders (-----) and non-responders (——) during treatment with tamoxifen (left) and tamoxifen, aminoglutethimide and danazol (right). Similar results were obtained with measurements of FSH.

DISCUSSION

The major measurable effect of tamoxifen was found to be suppression of gonadotrophins, and that of AGT was suppression of DHAS. It was for this reason that we examined these compounds in greater detail in relation to outcome of therapy. In general there was no difference in the extent to which hormones were suppressed by these agents in responders and non-responders to therapy. Responders to AGT alone appeared to show a more complete suppression of DHAS, but this was only demonstrated at the end of treatment, and could have been due to a longer course of therapy. Suppression of hormones appeared to continue as long as the course of therapy, even when the patient relapsed. Insufficient numbers of patients were treated with danazol to establish whether relapse with danazol therapy is due to failure of drug action, and further studies are currently in progress to determine whether this is so.

Relapse or non-response following endocrine therapy is therefore unlikely to be related to inadequate suppression of steroid hormones or gonadotrophins, though complete hormone suppression of oestrogens has not been demonstrated, mainly due to insufficiently sensitive assays. Thus, although suppression of oestradiol and oestrone are likely to be the most important effects of AGT therapy, the former is difficult to measure in postmenopausal women due to lack of adequate sensitivity, and the levels of oestrone are affected by imbalanced hepatic function, invalidating this assay in patients with hepatic metastases (M. Dowsett, personal communication). We have therefore measured drug action indirectly, by quantitating DHAS suppression which is a measure of the steroid C-20 and 22 hydroxylase-desmolase enzyme system. It appears that adequate suppression still operates on relapse. Similarly, the effect of tamoxifen on gonadotrophin concentrations is likely to be a secondary effect, since it is known that the major site of action of the drug is on RE (Jordan & Dowse, 1976). However, we have demonstrated that after tamoxifen therapy, the tumour is $RE^-$ (Taylor *et al.*, in press), suggesting that tamoxifen is still capable of binding to RE on relapse or non-response.

Relapse is therefore almost certainly due to an inherent property of cancer cells which enables them to regrow in an initially unfavourable endocrine environment. Further studies are being carried out to determine the other changes that may be responsible for relapse in these patients.

We thank Ms M. Abbott for technical help and the surgeons and radiotherapists of The Royal Marsden Hospital for permission to study their patients.

REFERENCES

COOMBES, R. C., DEARNLEY, D. P., HUMPHREYS, J., GAZET, J. C., FORD, H. T., NASH, A. G., MASHITER, K. & POWLES, J. T. (1980*a*) Danazol treatment for advanced breast cancer. *Cancer Treat. Rep.* **64**, 1073.

COOMBES, R. C., POWLES, T. J., GAZET, J.-C. & 4 others (1980*b*) Assessment of biochemical tests to screen for metastases in patients with breast cancer. Lancet, i, 296.

FRANCHIMONT, P. & CRAMILION, C. (1977) The effect of danazol on anterior pituitary function. *Fertil. Steril.*, **28**, 814.

GOLDER, M. P., PHILLIPS, E. A., FAHMY, D. R. & 4 others (1976) Plasma hormones in patients with advanced breast cancer treated with tamoxifen. *Eur. J. Cancer*, **12**, 719.

HAYWARD, J. L., CARBONE, P. P., HEUSON, J.-C., KUMAOKA, S., SEGALOFF, A. & RUBENS, R. D. (1977) Assessment of response to therapy in advanced breast cancer. *Cancer*, **39**, 1289.

JORDAN, V. C. & DOWSE, L. J. (1976) Tamoxifen as an anti-tumour agent: Effect on oestrogen binding. *J. Endocrinol.*, **68**, 297.

MCGUIRE, W. L., CARBONE, P. P. & VOLLMER, E. P. (1975) In *Estrogen Receptors in Human Breast Cancer*, New York: Raven Press p. 6.

SANTEN, R. J., SAMOJLIK, E., LIPTON, A. & 4 others (1977) Kinetic, hormonal and clinical studies with aminoglutethimide in breast cancer. *Cancer*, **39**, 2948.

SMITH, I. E., FITZHARRIS, B. M., MCKINNA, J. A. & 6 others (1978) Aminoglutethimide in treatment of metastatic breast carcinoma. *Lancet*, ii, 656.

TAYLOR, R. E., POWLES, T. J., HUMPHREYS, J. & 5 others (1982) Effects of endocrine therapy on steroid-receptor content of breast cancer. *Br. J. Cancer*, **45**, 80.

WARD, H. W. C. (1973) Antioestrogen therapy for breast cancer: A trial of tamoxifen at two dose levels. *Br. Med. J.*, i, 13.